# 400 mW narrow-linewidth Tm-doped silica fiber laser output near 1750nm with volume Bragg grating

**DOI:** 10.1038/srep12034

**Published:** 2015-07-13

**Authors:** Zhao Quan, Cunxiao Gao, Haitao Guo, Ning Wang, Xiaoxia Cui, Yantao Xu, Bo Peng, Wei Wei

**Affiliations:** 1 Institute of Advanced Materials, Nanjing University of Posts and Telecommunications, Nanjing 210023, P. R. China; 2 State Key Laboratory of Transient Optics and Photonics, Xi’an Institute of Optics and precision Mechanics, Chinese Academy of Science (CAS), Xi’an, Shanxi 710119, P. R. China

## Abstract

A type of Tm-doped silica fiber laser with narrow-linewidth and output wavelength near 1750 nm was firstly presented, by using a 1550 nm Er-doped fiber laser pump source and a volume Bragg grating (VBG). By means of a 15 cm Tm-doped fiber, a 400 mW continuous wave (cw) at ~1750 nm with FWHW of ~54 pm was generated, corresponding to an average slope efficiency up to 23.5% with respect to absorbed pump power. The influence of gain fiber length on the spectrum wavelength has been investigated in detail. All experimental results demonstrate that this fiber laser will be an effective and promising pump source for mid-IR laser output.

Tm-doped fiber lasers have attracted growing attention in numerous areas^1^ owing to their very broad transition linewidth over ~1.7 to 2.1 μm wavelength. Most valuably, they can act as pump sources for achieving 3.0 ~ 5.0 μm mid-infrared fiber lasers output at room-temperature, which have both national defense and commercial applications for imaging and remote sensing^2^,^3^. Unfortunately, the longest emission wavelength of infrared fiber laser is 3.95 μm, which is achieved in cryogenic Ho^3+^ ion doped ZBLAN glass fiber until now^4^. Extending to a longer wavelength such as 4.3 μm which is one of the most promising and valuable mid-infrared wavelength lasers especially in the applications of remote-sensing and national defense^5^, is a difficult but absorbing research subject. Early in 2008, R.S. Quimby and coworkers studied the numerical modeling of a continuous-wave fiber laser at 4.2–4.7 μm. In this case, a double-clad Dy^3+^ ions doped chalcogenide fiber was assumed, with direct pumping at 1.7 μm. It is heart-stirring that the calculated pump threshold is only 200 mW and the slope efficiency reaches 0.16 when the chalcogenide fiber loss is 1 dB/m^6^. It indicates that for 4.3 μm mid-infrared laser output, a gain fiber with high performance is prerequisite, but a practical laser pump source at 1.7 μm is necessary.

To our knowledge, the 1.7 μm region fiber lasers have been investigated before, but few are effective. The first report on single frequency 1735 nm distributed feedback (DFB) Tm-doped silica fiber laser was pumped with a Ti: sapphire laser at 790 nm, but only 1 mW maximum output and 0.2% slope efficiency were obtained^7^. Another typical work on Tm/Tb co-doped tunable fiber ring laser for 1716 nm lasing was pumped by a 1.21 μm laser diode. And the threshold power, maximum output power and slope efficiency were as low as 9 mW, 0.16 mW and 0.2%, respectively^8^. Although a tunable laser operating from 1723 to 1973 nm with multi-watts output power had been fabricated^9^, fiber core diameter of 20 μm and the lack of precision of simple bulk grating seriously influenced the linewidth and the modes of output laser. Recently, ultra-short wavelength of 1.7 μm regions has been developed by using FBG as wavelength selection, with wavelength tuning from 1720 nm to 1660 nm and fixed wavelength operation up to 12.6 W at 1726 nm^10^. Unfortunately, the laser parameters, such as the linedwidth of laser, the influence of gain fiber length on laser output and repeatability of in-house fabricated thulium doped active fiber weren’t discussed carefully.

Several popular ways for spectral narrowing are to use DFB or fiber Bragg gratings (FBGs), which offer high reflectivity with low insertion loss and free alignment. However, the disadvantages pointed out in Ref. 11 promote us to use a volume Bragg grating (VBG) for wavelength selection, such as hard to be tuned for wide range. Spectral narrowing and wavelength-tuning of Tm-doped fiber lasers using VBGs have been investigated and reported^9^,^11^,^12^,^13^. Up to 113 W of continuous-wave output at 1990 nm with a narrow linewidth of 2.2 pm and wavelength tunable from 1821 to 1930 nm with more than 52 W output power have been achieved respectively.

On the other hand, double-clad Tm-doped fiber lasers are usually cladding pumped by high-power multimode laser diodes operating at ~790 nm wavelength^14^. However, it is difficult to shift the range of Tm-doped fiber lasers to shorter wavelength region due to relatively long device length that is typical for cladding-pumped configuration^9^. An alternative approach is to pump directly into the upper level manifold (^3^F_4_) with a laser source at 1.55–1.62 μm. A good beam quality of Er-doped fiber lasers allows direct pumping into the core of the Tm-doped fiber, leading to the prospect of very high lasing efficiencies.

In this paper we report a narrow-linewidth Tm-doped fiber laser output at 1749.62 nm produced by core pump using a 1550 nm Er-doped fiber laser and a VBG. This laser was created by only ~15 cm length of Tm-doped fiber, corresponding to initial slope efficiency over 32.2% and average slope efficiency of 23.5% with respect to absorbed pump power. A maximum continuous wave (cw) output power of 400 mW and FWHW of ~54 pm was generated which, to the best of our knowledge, is the first report of cw laser output with both power of hundreds mW-level and narrow-linewidth at this wavelength.

## Results

**Figure 1:**
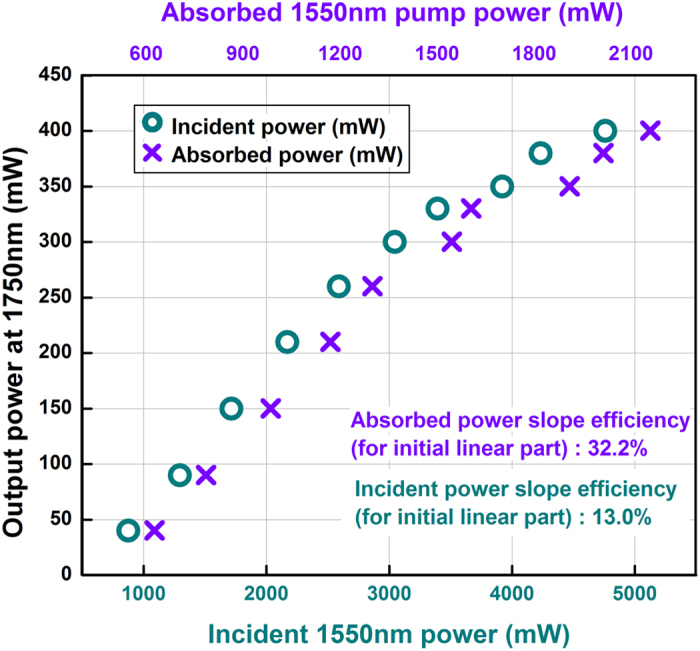
Evolution of output power at 1750 nm laser as linear function of absorbed (top scale) and incident (bottom scale) pump powers at 1550 nm.

Laser output power as a function of absorbed and incident pump power is illustrated in Fig. 1. It is found that the laser reached threshold at an absorbed pump power of about 600 mW and generated a maximum output power of 400 mW, corresponding to an average slope efficiency of 23.5% with respect to absorbed pump power.

**Figure 2:**
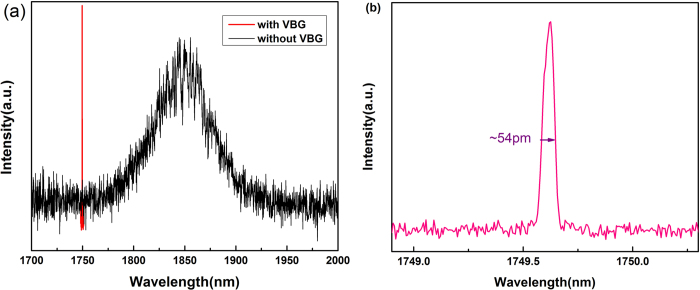
Laser output with VBG compared to fluorescence output without VBG (**a**) and optical spectrum of laser output at 1749.62 nm (**b**). Both of the input powers were 6 W.

Different spectra under two circumstances (with and without VBG) are measured in Fig. 2. It shows that the fluorescence without VBG has a bandwidth of ~200 nm centered at 1850 nm. The laser with VBG for wavelength selection with spectral FWHM of 54 pm laser spectrum centers at 1749.62 nm.

**Figure 3:**
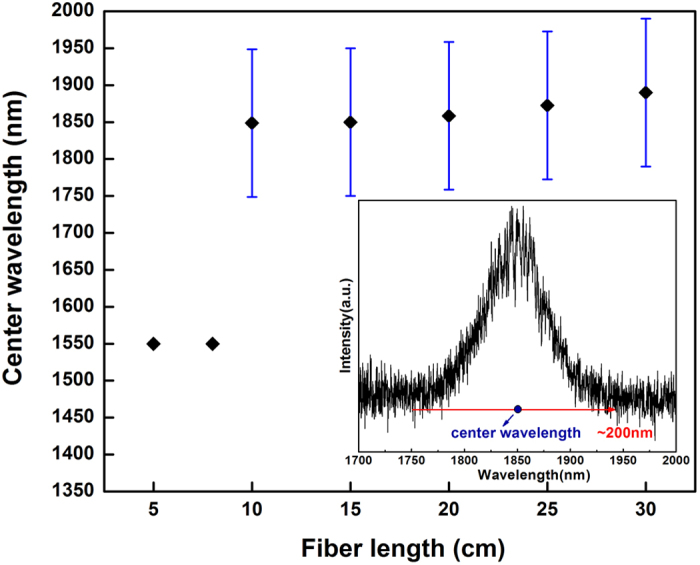
Relation between center wavelength of fluorescence spectrum and gain fiber length and typical fluorescence spectrum (inset).

**Figure 4:**
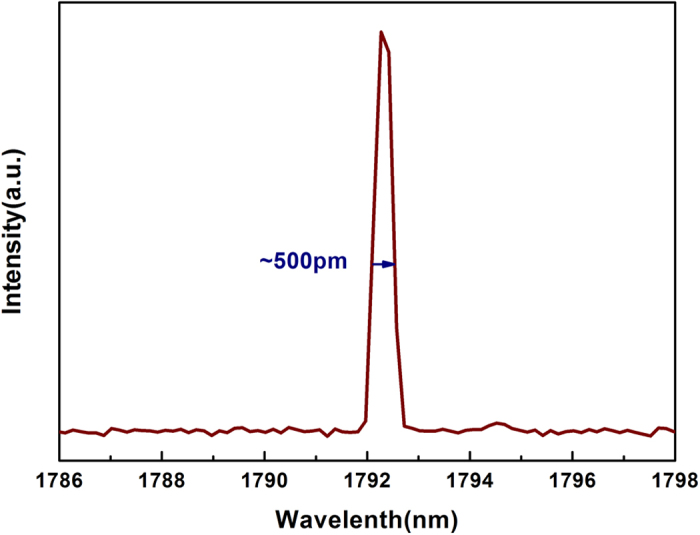
Typical laser spectrum at 1792.3 nm with gain fiber length of 30 cm (with FWHM of about 500 pm).

The relationship between the length of gain fiber and center wavelength in fluorescence spectrum is shown in Fig. 3 and a typical laser spectrum by using a long gain fiber length such as 30 cm is shown in Fig. 4. By decreasing the gain fiber length, the center wavelength of fluorescence spectrum shifts to short wavelength and a gain fiber with length of 15 cm has been chosen in this experiment for laser output at ~1750 nm. The availability of a narrow-linewidth laser source at ~1750 nm should be benefit for a range of applications, especially in 4.3 μm mid-infrared fiber laser production^5^.

## Discussion

The relatively high laser threshold of about 600 mW is attributed to high cavity loss, including collimation lens loss and splicing loss between the standard fiber and gain fiber. Meanwhile, due to the unbearably high temperature, higher pump power may result in fiber damage, which further lead to the low maximum output power of 400 mW (Fig. 1).

Moreover, it can be noted that the output power increases linearly with the absorbed pump power at low power levels with a higher slope efficiency of 32.2%, but begins to depress when the absorbed pump power exceeds 1.4 W. This regularity also shows in the relation of output power and incident pump power. Preliminary studies suggested that this behavior is related to the non-uniform temperature distribution along the fiber, which is further due to the non-uniform pump deposition density and quantum defect heating^15^. However, accurate physical mechanism and ways to alleviate the problem have not been figured out yet. After optimizing the water-cooled system, transmission of collimation lens and the process for fiber fusion splicing, effective improvement in the laser efficiency and output power could be expected.

Figure 2 compares the performance characteristics of the wavelength-locked narrow and Tm-doped fiber laser (with VBG) with that of a free running one (without VBG). The fluorescence without VBG is corresponding to the ^3^H_4_–^3^H_6_ transition of Tm^3+^ ion, which has an extraordinary broad emission bandwidth of ~200nm centered at 1850 nm. Low laser output power of 400 mW has been further decided by small emission section since 1750 nm locates at the edge of fluorescence spectrum. On the other hand, the laser with VBG for wavelength selection has a much narrower laser spectrum centered at 1749.62 nm which is highly fit to the VBG’s specified parameter 1749.6 nm. In contrast with specified parameter 1.6 nm, a spectral FWHM of 54 pm is obtained, which could be explained by mode competition due to the unflat-top reflectivity curve of the VBG, as a result of which, only the wavelength with lowest loss survived^16^. Moreover, laser cw output of shorter wavelength could be predicted by switching the parameters of gain Tm-doped fiber for wider fluorescence spectrum.

It should be noted that the length of gain fiber would influence the laser output. In this work, much shorter lengths of fiber were selected due to the relatively high core absorption efficiency. It is seen in Fig. 3 that the fluorescence spectra have broad emission bandwidth of ~200 nm and show no obvious change with different gain fiber length. But center wavelength shows blue shift phenomenon with decrease of gain fiber length. In addition, when the length of gain fiber is shorter than 10 cm, only the pump laser at 1550 nm can be detected. Fiber length changing from 15 to 30 cm makes greater influence on center wavelength of fluorescence spectrum. Only length of 10–15 cm regions could achieve ideal spectrum covering 1750 nm. Considering the factors of spectrum range, output power and the 1797.3 nm laser spectrum (produced by 30 cm gain fiber length, in Fig. 4), gain fiber length of 15 cm would be the best choice in this experiment.

## Methods

**Figure 5:**
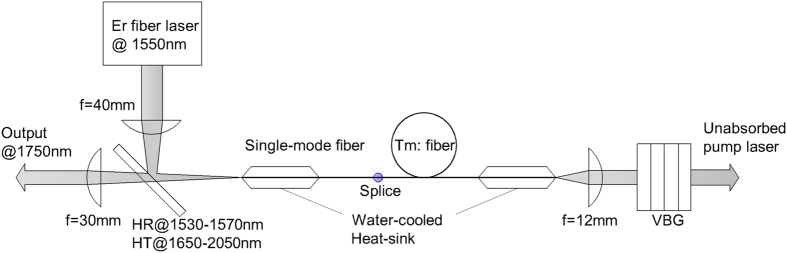
Schematic diagram of Tm-doped fiber laser core-pumped by a 1550 nm Er-doped fiber laser.

The experimental setup of Tm-doped fiber laser core-pumped by a 1550 nm Er-doped fiber laser is shown in Fig. 5. The double-clad single-mode fiber (Tm: fiber in Fig. 5) which had a pure Tm-doped silicate core of 6 μm diameter and ~0.23 NA was purchased from CorActive Corporation with a model of 100210-DCF-TM-6/125. Much short lengths of the fiber (10–25 cm) were used as active media due to the high core absorption efficiency. Pump light at 1550 nm was provided by an ELR-20-Y12 model Er-doped fiber laser from IPG LASER Corporation which could deliver a maximum cw output power of 20 W and emission bandwidth of 0.16 nm with beam quality factors (M^2^) of 1.07 and beam diameter (W) of 4.1 mm. To calculate couple and pump efficiency of gain fiber accurately, pump light was launched into the core of a standard single-clad single-mode pump delivery fiber (Single-mode in Fig. 1) through a 40 mm focal length lens and a 45° dichroic mirror with high transmission (>99%) at 1650–2050 nm and high reflectivity (>90%) at 1530–1570 nm. It is worth noting that in this experiment only ~35% of pump power was coupled into the single-mode fiber core mostly because of the much small fiber core diameter (only 6 μm) and not good ability of focusing by uncoated common lens. The gain fiber, whose free end was angle polished as greater than 8° was spliced to the output end of the single-clad fiber, and the loss of the splicing junction was measured to be 0.2 dB.

The laser resonator was formed on one side by Fresnel reflection provided by the perpendicular cleaved standard single-mode fiber end and on the other side by a 12 mm focal length collimating lens followed by a reflective VBG mounted in a copper heat sink. The VBG (OpticGrate Corporation) is 3.74 mm in thickness with an aperture of 8 mm × 6 mm and a relative diffraction efficiency of greater than 98% at a center wavelength of 1749.6 nm with FWHM of less than 1.6 nm. The fiber laser output was directly obtained by the same 45° dichroic mirror between the 40 mm focal length lens and perpendicular cleaved fiber end and collimated by a 30 mm focal length lens. Both end sections of the fiber were carefully embedded in special water-cooled V-groove aluminum heat sinks to facilitate heat removal and minimize the risk of thermally induced damage. What’s more, the spectrum of output was recorded by an optical spectrum analyzer with model of AQ6375 from YOKOGAWA Corporation.

## Additional Information

**How to cite this article**: Quan, Z. *et al.* 400 mW narrow-linewidth Tm-doped silica fiber laser output near 1750 nm with volume Bragg grating. *Sci. Rep.* **5**, 12034; doi: 10.1038/srep12034 (2015).
